# Study on Modulation Bandwidth and Light Extraction Efficiency of Flip-Chip Light-Emitting Diode with Photonic Crystals

**DOI:** 10.3390/mi10110767

**Published:** 2019-11-11

**Authors:** Hong Wang, Ming Zhong, Lijun Tan, Wei Shi, Quanbin Zhou

**Affiliations:** 1 Engineering Research Center for Optoelectronics of Guangdong Province, School of Physics and Optoelectronics, South China University of Technology, Guangzhou 510640, China; 201720127668@mail.scut.edu.cn (M.Z.); phsweet121@outlook.com (W.S.); 2 Guangdong Provincial Engineering Laboratory for Wide Bandgap Semiconductor Materials and Devices, School of Electronics and Information Engineering, South China University of Technology, Guangzhou 510640, China; eeljtan@mail.scut.edu.cn (L.T.); zhouquanbin86@163.com (Q.Z.); 3 Zhongshan Institute of Modern Industrial Technology, South China University of Technology, Zhongshan 528437, China

**Keywords:** visible light communication, photonic crystals, flip-chip LED, Purcell effect, light extraction efficiency

## Abstract

In this study, the photonic crystal structure is employed to increase both the light extraction efficiency and the modulation bandwidth of flip-chip GaN-based light-emitting diodes (LEDs). The finite difference time domain method is utilized to investigate the influence of structure of photonic crystals on the Purcell factor and light extraction efficiency of flip-chip GaN-based LEDs. Simulation results show that the modulation bandwidth is estimated to be 202 MHz at current densities of 1000 A/cm^2^. The experimental result of modulation bandwidth is in accord with the simulation. The optical f-3dB of the device achieves 212 MHz at current densities of 1000 A/cm^2^ and up to 285 MHz at current densities of 2000 A/cm^2^. This design of photonic crystal flip-chip LED has the potential for applications in high-frequency visible light communication.

## 1. Introduction

Visible light communication, as a communication solution to alleviate the shortage of spectrum resources, is at the frontier of technology and the hotspot of research. GaN-based light-emitting diode (LED) chips are key devices for visible light communications. However, the narrow bandwidth of commercial LED chips, which is in the range of 20–30 MHz, limits the overall bandwidth of visible light communication systems [1,2]. The modulation bandwidth of an LED chip is limited by the resistance–capacitance (RC) time constant and carrier spontaneous emission rate [3]. Therefore, the methods to increase the modulation bandwidth mainly include decreasing the RC time constant [4,5,6] and the carrier lifetime [7,8]. Many studies have shown that the wavelength-sized cavity can change the local density of optical states (LDOS) and increase the spontaneous emission rate [9]. In 1946, Purcell proved that the spontaneous radiation rate of the cavity can be changed by the Purcell factor [10]. Nanostructures, such as resonant cavities, surface plasmon, and photonic crystals, can affect the Purcell factor, thereby increasing the spontaneous emission rate of carrier and the modulation bandwidth. Although there are many literatures studying photonic crystal structure to improve the modulation bandwidth of common LED [7,11,12], the literature about flip-chip LEDs with photonic crystals on the bottom is still rarely explored.

In this work, both modulation bandwidth and light extraction efficiency (LEE) of flip-chip LEDs with photonic crystals are numerically investigated by using a 3D finite-difference time-domain (FDTD) method based on Yee’s algorithm with a perfectly matched layer (PML) boundary condition [13]. Three structural parameters of photonic crystals including period, height, and duty cycle were compared to study the effect of photonic crystals on the modulation bandwidth and LEE of a flip-chip LED. By optimizing the photonic crystal structure, we designed a structure that could simultaneously improve the modulation bandwidth and light extraction efficiency of the flip-chip GaN-based LEDs. The experimental result shows that photonic crystal structure can improve the modulation bandwidth due to the enhancement of the Purcell factor and the reduction of nonradiative lifetime.

## 2. Materials and Methods 

**Figure 1:**
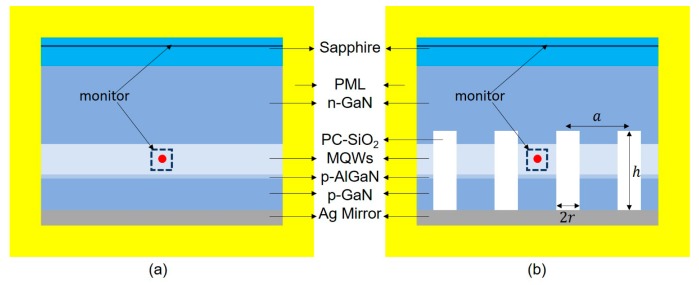
Model of (**a**) the conventional planar flip-chip light-emitting diodes (LED) and (**b**) the flip-chip LED with photonic crystals. Red dots inside multiple quantum wells (MQWs) represent the position of the dipole source.

Figure 1 shows the model of a conventional planar flip-chip LED (FCLED) and flip-chip LED with photonic crystals (PC-FCLED). The lateral dimensions of the computational domain were set to 7 µm × 7 µm × 7 µm with a mesh of 2 nm × 2 nm × 2 nm. In FCLED, the structure consisted of 3 mm sapphire substrate (extending beyond the simulation volume), a 5000 nm n-GaN layer, a 120 nm multiple quantum wells (MQWs) layer, a 20 nm p-AlGaN layer, a 110 nm p-GaN layer, and a 100 nm Ag mirror layer. The material parameters in the simulation were mostly from reference [14]. Because the radiation in the quantum well was mainly in transverse electric (TE) mode, the light source was set to the TE mode dipole source [15]. The dipole source was placed in the center of the MQWs layer of the plane of the sectional view. The wavelength of the light source was 460 nm. Six power monitors were placed near the light source on the six faces for detecting total power from the dipole. Considering the large thickness of the sapphire substrate, a monitor was placed in the sapphire substrate 1 µm from n-GaN layer, and the power exiting in the air that far away from the source was calculated by way of far-field projection. The boundary conditions were set as a perfectly matched layer (PML). The top of the FDTD simulation area was located inside the sapphire substrate, and the bottom was placed inside the mirror to ignore the cavity effect it generated [16]. As for PC-FCLED, the model was similar to FCLED except for the photonic crystals. The square lattice photonic crystals with an array of SiO_2_ rods were placed in p-GaN of PC-FCLED, as shown in Figure 1b. The period, height, and radius of photonic crystals were defined as *a*, *h*, and *r*, respectively. The duty cycle of photonic crystals was 2*r*/*a*. The LEE was defined as the ratio of the power exiting the structure (flux calculated by way of far-field projection) to the total power generated by the dipole inside the active region (flux through the small box in Figure 1).

In 2009, Lau et al. deduced the approximate bandwidth of a nanocavity LED [17]:

(1) $$f_{3dB}\approx \frac{1}{2\pi }\frac{1}{\sqrt{\tau_{p}^{2}+\tau_{eff}^{2}}}$$

where $\tau_{eff}$ is the spontaneous radiation lifetime reduced by the Purcell effect, and $\tau_{p}$ is the photon lifetime. For cavities with quality factors less than a few hundred, $\tau_{p}$ is much lower than the overall lifetime, and $f_{3dB}$ mainly depends on $\tau_{eff}$ [18]. The relationship between $f_{3dB}$ and $\tau_{eff}$ is as follows:

(2) $$f_{3dB}\approx \frac{1}{2\pi }\frac{1}{\tau_{eff}}$$

(3) $$\frac{1}{\tau_{eff}}=\frac{F}{\tau_{r}}+\frac{1}{\tau_{nr}}$$

where *F* is the Purcell factor. Then,

(4) $$f_{3dB}\approx \frac{1}{2\pi }\left[\frac{F}{\tau_{r}}+\frac{1}{\tau_{nr}}\right]$$

For polar c-plane InGaN/GaN LEDs at current densities of 1000 A/cm^2^, David et al. derived the carrier lifetime [19]: $\tau_{r}=3\;\mathrm{ns}$; $\tau_{nr}=1.5\;\mathrm{ns}$.

Thus, the modulation bandwidth of the LED can be approximated by simulating the Purcell factor.

## 3. Results and Discussion 

**Figure 2:**
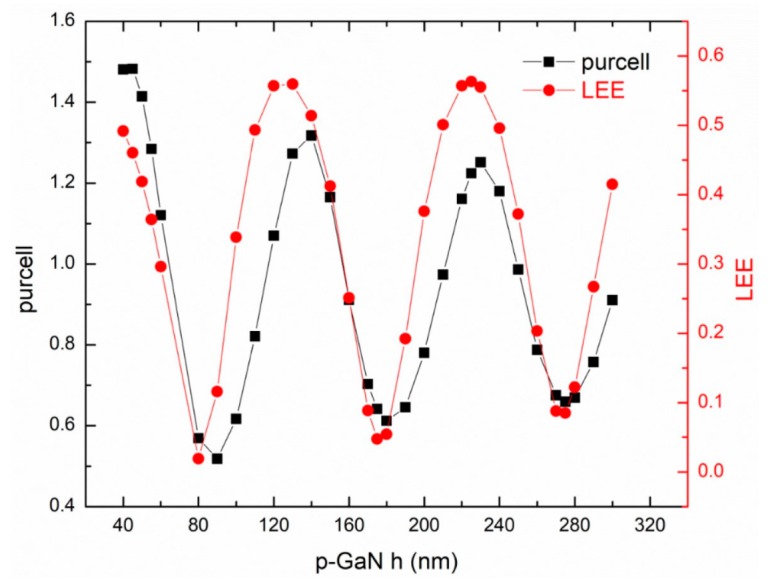
The effect of p-GaN thickness on the Purcell factor and light extraction efficiency (LEE) for flip-chip LED (FCLED).

Figure 2 shows the effect of p-GaN layer thickness on the Purcell factor and LEE for FCLED. Both the Purcell factor and LEE show a trend of sinusoidal function oscillating when the p-GaN layer thickness increases. Moreover, the Purcell factor tends to oscillate and attenuate, and the LEE exhibits a perfect periodic oscillation. The trends of the Purcell factor and the LEE are almost the same, which is expected to increase the LEE and the modulation bandwidth at the same time. The Purcell factor takes a peak point of 1.48 when the p-GaN thickness is 45 nm, and then takes a peak point at 140 nm and 230 nm. The periodicity of the Purcell factor with p-GaN thickness is 90 nm, which is almost the same as half the wavelength of the light source inside the p-GaN material. When the p-GaN thickness is 140 nm, the Purcell factor is 1.32 and the LEE is 51.4%.

**Figure 3:**
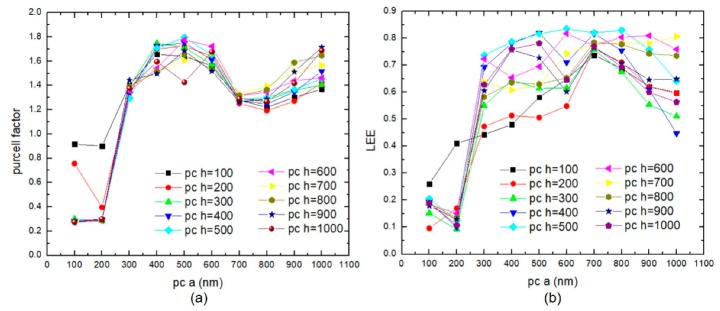
The trend of (**a**) Purcell factor and (**b**) LEE varying with the period and height of the photonic crystals when the duty cycle is 0.5.

Figure 3 shows the trend of the Purcell factor and LEE varying with the period and height of the photonic crystals when the duty cycle is 0.5. When the period of photonic crystals is greater than 200 nm, the Purcell factor is above 1.2. When the period and height of photonic crystals are both 500 nm, the Purcell factor is 1.8, which is better than the results of similar simulations [14,16]. Because the small period photonic crystal structure (the period 100 and 200 nm) cannot provide enough mode volume to the lower-order mode for affecting the radiative recombination, the large period photonic crystal structure in the proper duty cycle has a chance to couple with the lower-order mode with a higher spontaneous emission rate, which leads to the high Purcell factor [20]. Photonic crystal structures with different heights have a larger Purcell factor in the photonic crystal period of 400–600 nm. When the photonic crystals period is greater than 250 nm, the LEE is above 40%. The small period photonic crystal structure cannot maintain enough emission light in the extraction cone, and the emission light is almost too hard to go through the surface [21].

**Figure 4:**
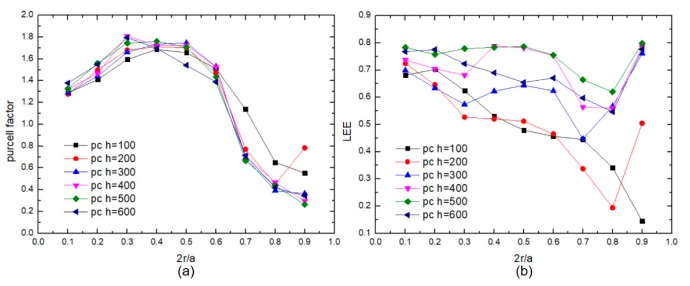
The tendency of (**a**) the Purcell factor and (**b**) LEE changing with the duty cycle and height of the photonic crystals when the photonic crystal period is fixed at 400 nm.

Figure 4a shows the tendency of the Purcell factor and LEE to change with the duty cycle and height of the photonic crystals when the photonic crystal period is fixed at 400 nm. The Purcell factor increases first and then decreases with the duty cycle increasing. The Purcell factor is 1.81 when the height and duty cycle of photonic crystals is 400 nm and 0.3, respectively. When the duty cycle is in the range of 0.1–0.6, the Purcell factors with different photonic crystal heights are all greater than 1.2. This is because photonic crystal height cannot strongly change mode volume, compared with duty cycle and period of photonic crystals. When the duty cycle is larger than 0.8, there is not enough mode volume for the lower-order mode to achieve a high Purcell factor due to the reduction of the active region area. As shown in Figure 4b, when the photonic crystal period is fixed at 400 nm, the LEE varies with the duty cycle and height of the photonic crystals. When the photonic crystals are deep into the active region, optical modes interact with the photonic crystals and are diffracted to escape, which increases the LEE [22]. When the photonic crystal height is 400 nm and the duty cycle is 0.3, the Purcell factor and LEE of PC-FCLED are 1.81 and 68%, which is 37% and 32.3% higher than those of a conventional planar FCLED, respectively.

These results show that the PC-FCLED has a better LEE and Purcell factor than FCLED. When the photonic crystals are deep into the active region, the influence of the height of the photonic crystals on the Purcell factor decreases. When the photonic crystal period is more than 300 nm, the Purcell factor and LEE are better. The duty cycle, which is between 0.1–0.6, is more conducive to the improvement of the overall performance. With the decrease of the radiation carrier lifetime and the fast photon accumulation effect in the specific photonic band gap, the PC-FCLED obtains both high output optical power and large modulation bandwidth [20]. Therefore, the modulation bandwidth and LEE are increased simultaneously due to the Purcell effect in PC-FCLED. According to the above derivation and results, the trend of the modulation bandwidth of PC-FCLED changing with the Purcell factor can be obtained. When the Purcell factor is 1.81, the bandwidth is about 202 MHz at current densities of 1000 A/cm^2^.

**Figure 5:**
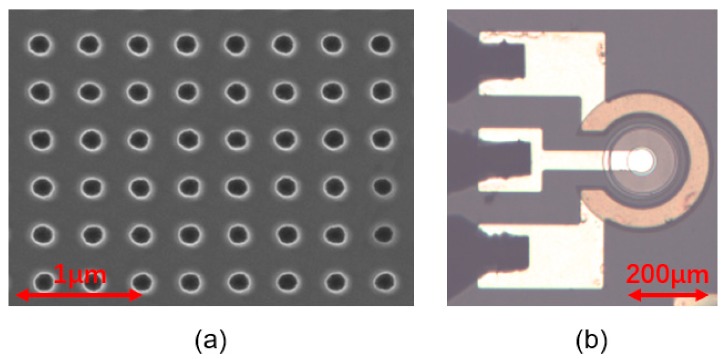
(**a**) The scanning electron microscopy (SEM) images of the hole-patterned photonic crystal structure. (**b**) Top view of PC90 LED chip with mesa radius of 90 µm.

The optimized photonic crystal structure of the simulation was obtained for subsequent experimentation. The studied PC-FCLEDs were prepared by using the epitaxial wafers with a peak wavelength of 460 nm in the experiment. The LED structure consisted of a 3 µm-thick undoped GaN layer, a 1.8 µm-thick n-type GaN layer, 9 periods of InGaN/GaN MQWs, and a 140 nm-thick p-GaN layer. Then, the square lattice photonic crystal was formed in the p-GaN layer and MQWs by nanoimprint lithography and inductively coupled plasma (ICP). Figure 5a shows the scanning electron microscopy (SEM) images of the photonic crystal structure after the patterns were etched with a photonic crystal period of 400 nm, hole depths of 400 nm, and duty cycle, which is 0.3. A Spin-On-Glass (SOG) layer was deposited on the surface of p-GaN and then annealed at 400 °C for 30 min by rapid thermal annealing to fill in the hole. Subsequently, the mesa regions with a depth of 1.3 µm were transferred to the LED wafer using ICP etching. Ag and TiW metal layers were deposited on the top surface of the LED wafer, and a 1 µm-thick SiN layer was deposited to mesa sidewalls by plasma-enhanced chemical vapor deposition. Finally, Cr/Al/Ti/Au (50/800/200/200 nm) multilayer metals were sequentially deposited on the top of Ag and TiW layers and the n-GaN layer to act as the p- and n-electrodes by electron beam evaporation, respectively. The nomenclature of PC-FCLEDs with different mesa radii were PC30, PC60, and PC90. FCLEDs were also fabricated using the same procedure as the PC-FCLEDs, but without photonic crystals.

**Figure 6:**
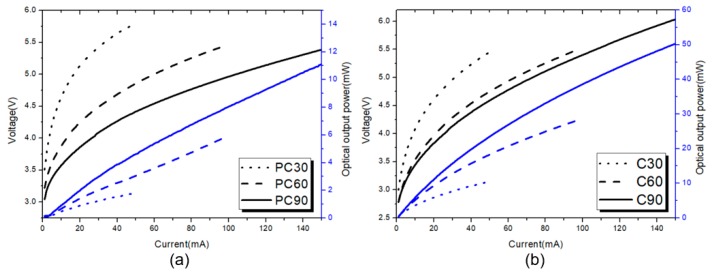
The optical output power and forward current–voltage (P-I-V) curves of (**a**) flip-chip LEDs with photonic crystals (PC-FCLEDs) and (**b**) FCLEDs of different mesa radii.

Figure 6 represents the optical output power and forward current–voltage (P-I-V) characteristics of the PC-FCLEDs and the FCLEDs. The P-I curves show that the optical output power of PC-FCLED is smaller than that of the FCLED with the same mesa size. This is because, even though the photonic crystal helps light extraction, the effective light emitting area of the PC-FCLED is smaller than the FCLED, and the damage of p-GaN from the ICP procedure will strongly reduce carrier recombination efficiency.

**Figure 7:**
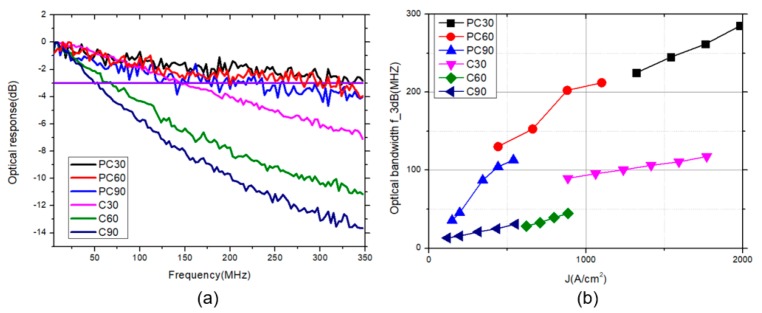
(**a**) Frequency responses of PC-FCLEDs and FCLEDs at an injection current of 50 mA, 100 mA, and 150 mA with mesa radii of 30 µm, 60 µm, and 90 µm, respectively. (**b**) Optical bandwidth f−3dB of PC-FCLEDs and FCLEDs at various current densities.

The optical modulation bandwidth (f-3dB) versus current density relation of the PC-FCLEDs and FCLEDs with three different radii are shown in Figure 7. The optical f-3dB in PC-FCLEDs is much higher than that of the FCLEDs due to the smaller carrier lifetime. At a 1000 A/cm^2^ injected current density, the optical f-3dB of PC60 is 212 MHz, which is almost in accord with the simulation result of 202 MHz. As the mesa size of the device decreases, the current density can be improved to achieve a higher modulation bandwidth. When the injected current density is 2000 A/cm^2^, the optical f−3dB of PC30 increases up to 285 MHz. However, at a 1000 A/cm^2^ injected current density, the optical f−3dB of C30 is 90 MHz, which is lower than the simulation result of 176 MHz. This means that carrier lifetimes of FCLEDs are lower than the carrier lifetimes quoted, and there is another mechanism here that leads to the decrease of the carrier lifetimes of PC-FCLEDs. Therefore, the Purcell factor plays a certain role in the increase of modulation bandwidth, but cannot completely determine its implementation. In the ICP procedure for etching photonic crystal structure, many defect sites are produced in the p-GaN layer and MQWs. The increase of defect density causes a reduction of the radiative efficiency and nonradiative lifetime [23], which leads to further increasing the modulation bandwidth and reducing optical output power.

## 4. Conclusions

We investigated the Purcell factor and LEE of PC-FCLED using the FDTD method. Compared with the FCLED, the PC-FCLED shows great enhancement in the Purcell factor and LEE. When the height and duty cycle of photonic crystals is 400 nm and 0.3, the Purcell factor and LEE of PC-FCLED are 1.81 and 68%, which is 37% and 68% higher than that of FCLED, respectively. When the Purcell factor is 1.81, the modulation bandwidth is 202 MHz at current densities of 1000 A/cm^2^. We constructed the devices, and the experiment results of the modulation bandwidth achieved the desired level of simulation because of the reduction of nonradiative lifetime and increase of the Purcell factor. An optical f-3dB of 285 MHz was obtained in the PC-FCLED with a mesa radius of 30 µm at a current density of 2000 A/cm^2^. The PC-FCLED revealed the potential for visible light communication due to its high modulation bandwidth.
